# Introducing R as a smart version of calculators enables beginners to explore it on their own

**DOI:** 10.12688/f1000research.54685.1

**Published:** 2021-08-26

**Authors:** Krishna Choudhary, Alexander R. Pico

**Affiliations:** 1 Institute of Data Science and Biotechnology, Gladstone Institutes, San Francisco, CA, 94158, USA; 2 Diabetes Center, University of California San Francisco, San Francisco, CA, 94143, USA

**Keywords:** students, data science training, learn programming, R

## Abstract

Rapid technological advances in the past decades have enabled molecular biologists to generate large-scale and complex data with affordable resource investments, or obtain such data from public repositories. Yet, many graduate students, postdoctoral scholars, and senior researchers in the biosciences find themselves ill-equipped to analyze large-scale data. Global surveys have revealed that active researchers prefer short training workshops to fill their skill gaps. In this article, we focus on the challenge of delivering a short data analysis workshop to absolute beginners in computer programming. We propose that introducing R or other programming languages for data analysis as smart versions of calculators can help lower the communication barrier with absolute beginners. We describe this comparison with a few analogies and hope that other instructors will find them useful. We utilized these in our four-hour long training workshops involving participatory live coding, which we delivered in person and via videoconferencing. Anecdotal evidence suggests that our exposition made R programming seem easy and enabled beginners to explore it on their own.

## Introduction

*“The stepwise introduction into R from the simple explanation of the system as a calculator was very approachable and I felt that I was able to move into the system with a lot less trepidation and more curiosity to explore on my own.”*— Anonymous feedback from an attendee of our R workshop in March, 2019

The last two decades have witnessed relentless developments in high-throughput technologies for DNA sequencing, proteomics, imaging, and a variety of sequencing-based biochemical assays. ^1- 5^ Easy availability of large-scale data from public databases and an ever-accelerating pace of data generation facilitated by continued decreases in costs have revolutionized the way biological research is conducted. ^6- 8^ Now, large-scale data plays a central role in biological research. Yet, multiple surveys with participants from around the globe have recognized a lack of basic data science skills among graduate student researchers (pursuing Masters or doctoral degree programs), postdoctoral researchers, senior academics, technical staff, and industry researchers (henceforth, collectively called *postgraduate learners*). ^9^ While some undergraduate bioscience degree programs have adapted by training students in data science skills, ^10- 12^ basic computational and statistical skills are still relatively rare in the bioscience curricula. ^9^ This means that students progress to advanced research without basic data science skills, thereby adding to an already large number of postgraduate learners lacking these skills. To help these learners, many universities have launched generic postgraduate degree programs in data sciences with or without a focus on biological applications. However, global surveys have revealed that the majority of postgraduate learners prefer short face-to-face training workshops. ^9^ To serve this demand, a number of institutions around the world are offering workshops in core data science skills. ^13- 15^ This article focuses on the design and content of such workshops with an emphasis on the needs and expectations of postgraduate learners who may be *absolute beginners* in programmatic analysis.

The surveys that we referred to above have identified challenges in delivering high-value content in short workshops. ^9^ They revealed that most postgraduate learners seek training when they have already collected data for their ongoing projects. Attwood *et al.* noted that the retention of skills acquired to perform specific analyses at a particular time tends to be poor. Hence, trainees often need to attend the same workshops again. Furthermore, if the trainees cannot apply their newly acquired skills to performing research in their own time in the weeks and months since training, their confidence may be diminished. Yet, we found that most introductory workshops for absolute beginners in, say, R or Python programming often focus on basic operations such as reading data, basic arithmetic, subsetting tables, basic plots, etc. In our assessment, these operations are essential to analysis and should be a part of an introductory workshop, but they alone do not generate a feeling of confidence that one could derive practical utility from one short workshop. Given that some of the trainees may not have time nor motivation for a second workshop, it is important that the very first workshop yield real value for the trainees, and more importantly, equip them with a mental framework to explore computational analysis on their own. Additionally, postgraduate learners may possess higher levels of experience in research and teaching than undergraduates, which makes them harder to persuade. For example, if undergraduates are told in a class that they need to work with a programming language to analyze data instead of Excel spreadsheets, they may commit to learning without asking why. In our experience, postgraduate learners may not vocally demand a justification, but at the same time, they may not be sold on programming without one. Even for undergraduates, this seems to be an unreasonable expectation. Effective workshop designs are required to train the postgraduate learners and can enhance the quality and quantity of biological research. ^9, 13^

In this article, we propose that introducing programming languages for data analysis as smart versions of calculators can enhance the effectiveness of introductory workshops. Given the popularity of R language for statistical analysis of biological data, we use R programming as a means to elucidate our proposition. Our view is that for data analysis, R is an evolved form of traditional calculators in the same way that smartphones are an evolved form of earlier-generation mobile phones. The evolution in both cases has been made possible by adoption of a new and intuitive way to interface with technology—touch interface over buttons in the case of smartphones, and programmable functions over push-button functions of calculators in the case of R programming. In training someone on how to use a smartphone, even if the instructor were to show how to use a specific application on a smartphone, the transferable learning for independent smartphone use by the trainee would be an implicit understanding of the interface itself and not the knowledge of options in menus of the application that they might have been shown. Similarly, in an introductory R workshop, if absolute beginners are given an understanding of design elements of R using a set of commands as a means but not the primary intended deliverable, it might enable them to independently explore R in the weeks after the workshops. In our workshops, this design philosophy has helped us dispel prior beliefs of absolute beginners that they need to memorize a large number of R commands before deriving practical use from R. Instead, with a knowledge of how to interface with the “smart” version of calculators that we view R to be, intuition underlying its design, and hands-on analysis experience that we were able to integrate in one 4-hour-long workshop, we found that our trainees felt equipped to explore R for their practical research purposes. Further, we could provide trainees with specific use cases that they could make a part of their research process. Our approach helped us shift the focus to statistical concepts in later hands-on analysis workshops while the code took a backseat. Here, we utilize analogies with commonly known objects to explain the intuition underlying key elements of the R system and present it as a “smart” version of calculators. Additionally, we propose how trainees could begin to integrate R in their research after an introductory workshop. Volunteered comments in anonymous feedback forms (collected online) and direct feedback from researchers in our local community indicated that for at least some of the trainees, our workshops achieved the desired outcome of enabling beginners to explore R on their own. Borrowing ideas from our work might yield beneficial outcomes in other introductory workshops on programmatic data analysis.

## Highlighting the analogy between calculators and R can enhance the effectiveness of training

Since the dawn of civilization, humanity has needed to perform quantitative analyses for a variety of purposes, e.g. commerce and taxation. To serve this need, quantitative notation systems, tools and devices have been under development throughout our history. ^16^ Calculators for basic arithmetic operations and R for statistical analyses are examples of such devices. Most postgraduate learners almost certainly have prior experience with calculators. This makes them a useful analogous prior knowledge that can serve as a bridge to the world of programming that is unknown to absolute beginners (see ^17- 20^ for discussion on the use of analogies in teaching). We have leveraged this analogy to make the following points in our workshops.

**Figure 1.**
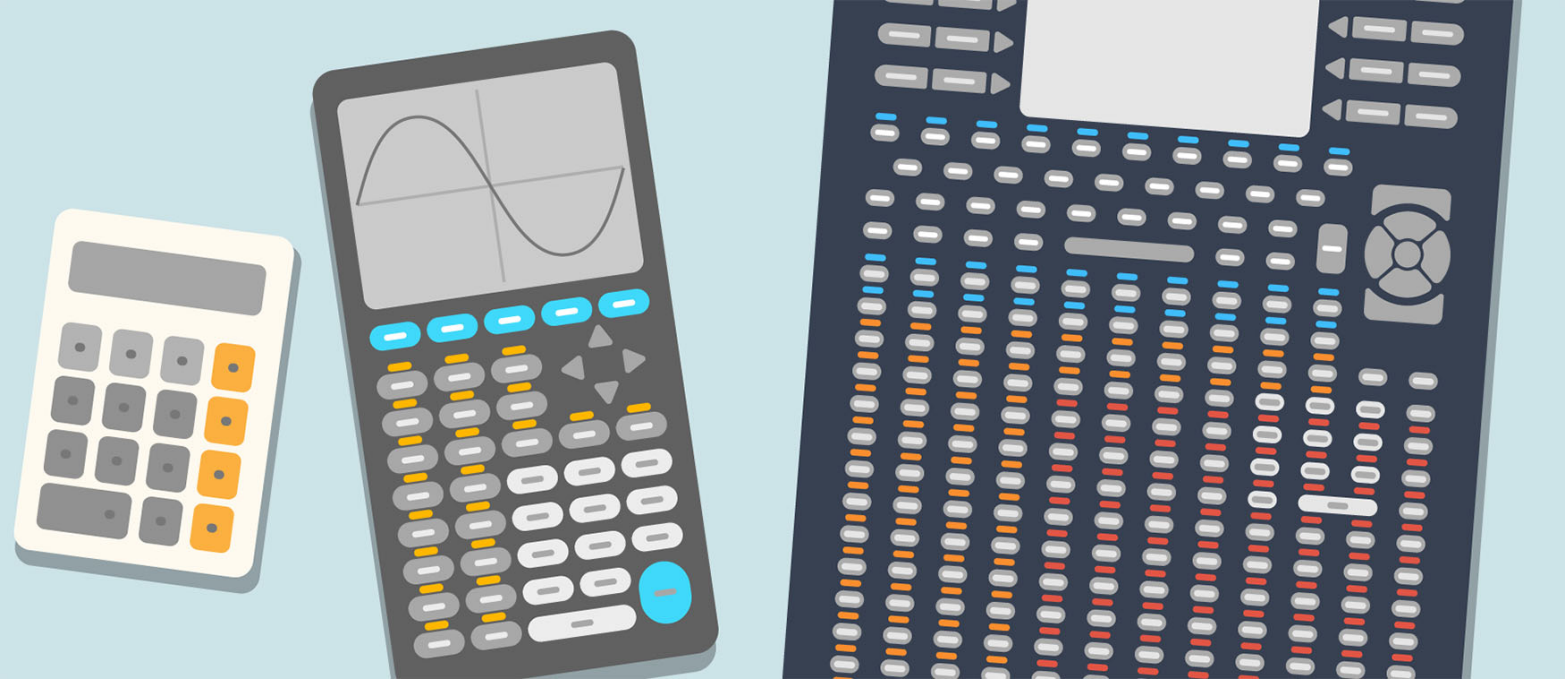
R is a smart version of calculators. Like the traditional hand-held calculators, R is a device for quantitative computations. However, R supports a myriad of quantitative operations that are required for modern biosciences. If the order of 100,000 R functions were to be accessed via push button keys, i.e., the same way as traditional calculators—one key per function, the device would be unwieldy. A programming language is a logical way to interact with a “smart calculator”.

1. R uses programmable functions, which are like the push-button functions on a calculator, only much more customizable and dynamic. This supports the myriad quantitative operations required for modern biosciences that far outnumber the basic arithmetic operations supported by calculators. An illustration such as in Figure 1 makes programming appear as a logical and more practical way to do statistical analyses.
2. An absolute beginner may feel that they need to be good at statistics to derive any use out of R. This can result in loss of motivation and prevent them from exploring R on their own. We used the analogy with calculators to convey that this is not the case. For example, the square root function on a calculator can be used effectively, even if one is not skilled at performing the operation mentally or manually on paper. Similarly, postgraduate learners may already have a high-level understanding of the tasks that they need to implement from studying research literature, e.g., clustering of data points, making volcano plots given fold changes in gene expression and associated p-values, etc. Programmable functions in R are but an analogue of the buttons comprising a calculator’s keypad. To learn to “push the buttons” of the R system is an achievable goal in a short time frame, especially because R is designed to be a “smart” version of calculators (see the next section).
3. To get good at arithmetic, it is important to know the method by which one adds, subtracts, multiplies, or divides by hand. Similarly, one must strive to understand what the algorithms implemented in R functions are doing. Learning to program in R is a good starting point towards that goal. Further, as we discuss later, there are ways in which one can achieve synergy by combining their beginner-level knowledge of programming with the statistical know-how of their collaborators and/or supervisors.

## R is a smart version of calculators

Analogy between R and calculators stretches beyond them both being devices for quantitative computations. In fact, R may be viewed as a “smart” version of calculators. Among its many improved features, the version advance has been made possible by adoption of a new interface (programmable functions instead of push-button functions), utilization of internet connectivity (e.g., internet-enabled package installations for new functions), support to save analysis sessions to secondary memory, availability of functions for diverse applications, and support for literate programming. As described below, in our workshops, we presented some of the key elements that constitute the R system as logical designs to upgrade calculators to a “smart” version.

**New interface via programming.** Calculators perform only a few basic arithmetic operations accessible via push-button functions. In contrast, R is designed to perform a myriad of tasks. To enable this advance, R users must spell out the name of the functions that would otherwise be button labels if R functions were to be accessed via a keypad. Further, simple arithmetic performed by the push-button functions of calculators accept one or a few numeric inputs. In contrast, programmable functions in R can take a variety of inputs. These are passed to functions as named arguments enclosed in parentheses. The function and argument names are conventionally meaningful, utilize common language and context-specific words, and follow syntax patterns. These features make them easy to look up and remember. Hence, programmable functions have been the preferred way to interface with R.

**Internet-enabled updates to available options via packages.** Software updates by installations of files obtained from internet is a defining feature of smart devices. A package is the R equivalent of *apps* on a smartphone. To understand why this is a logical feature of a smart calculator, it is helpful to consider the logic underlying smartphone apps, which is something that all postgraduate learners are familiar with. An app store is a digital platform that distributes a large number of apps— each app serving a specific need. If all these apps were pre-installed on a smartphone, they would occupy a lot of storage. Hence, in the age of internet connectivity, the smart solution is to have a single platform from which apps are available on demand. Similar solution exists in programming languages such as R. Users can install R packages from online repositories such as CRAN ^21^ and Bioconductor. ^22^ The packages provide access to a set of functions that have been written to facilitate a specific analysis, e.g., *edgeR* for differential expression analysis of RNA-seq data. There may be multiple packages that serve the same purpose, e.g., *edgeR* and *DEseq2*, which is analogous to there being multiple smartphone apps for the same purpose, e.g., Google Maps and Waze for navigation. Each R package may have its own distinguishing feature or selling point, which might make it more suitable for a particular use case. From an R user’s perspective, when faced with the task of processing biological data, a good starting point might be to do a literature search for current best practices in analysis of the kind of data they have, which would typically result in recommendations for R packages. Alternatively, the user might find such information in the methods sections of articles that may have used such data, or study literature and surf online discussion forums for reviews of different packages. These steps are analogous to those one might take in picking a smartphone app for their needs. It is important to hit these points in introductory workshops because we found that absolute beginners harbor a misconception that advanced bioinformaticians are always building methods for analysis from scratch for every task.

**Figure 2.**
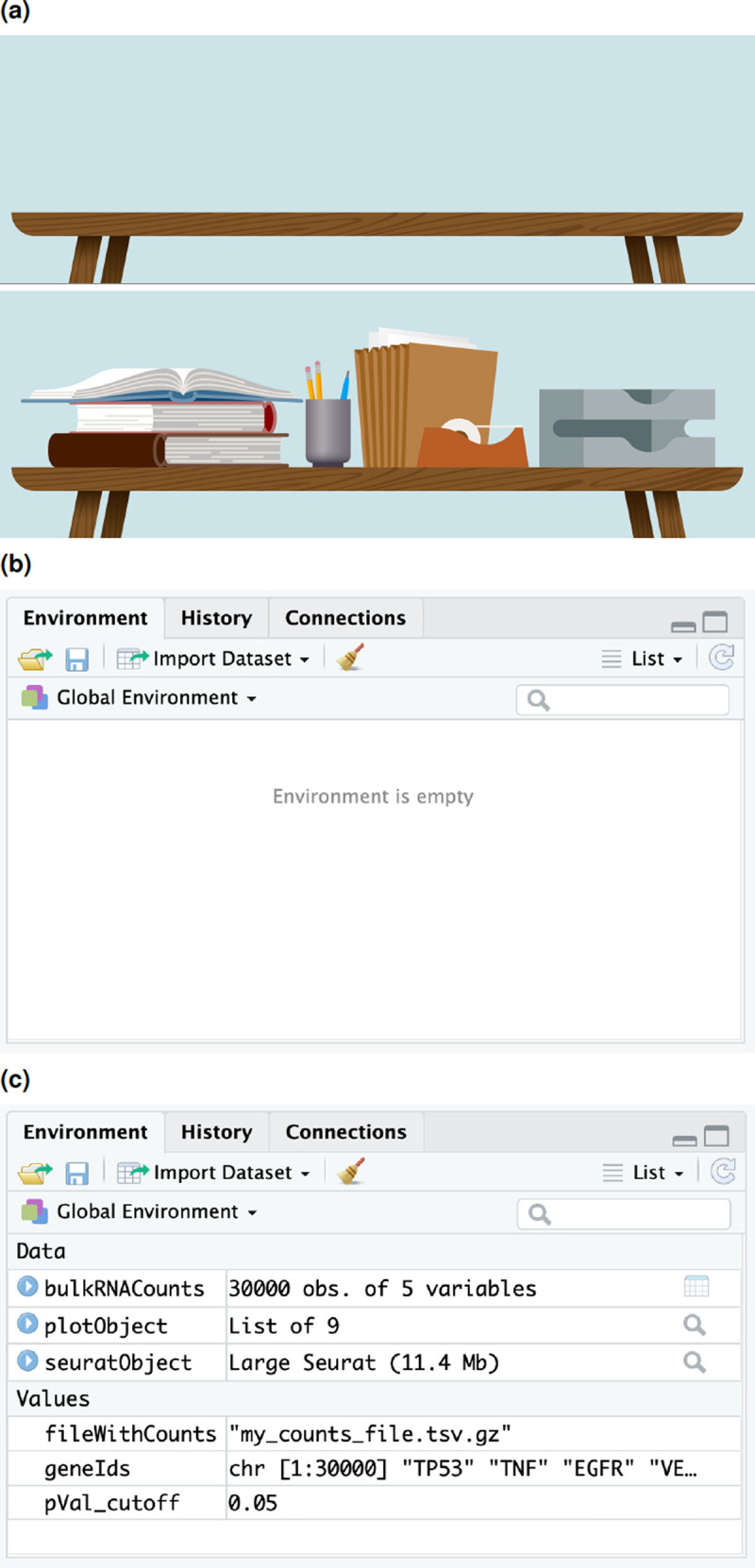
R environment has a physical analogue. (a,b) An empty R environment is analogous to an empty desk at the start of analysis. (a,c) As analysis progresses, objects with data are stored in the current environment.

**Secondary memory for long-term storage.** A typical calculator lacks secondary memory, and hence, does not support saving work sessions. In contrast, the modern devices that support access to R, typically provide ample secondary storage and access to large-scale remote storage via internet. In turn, R supports saving outputs of computations in a diverse range of file formats including image file formats and biological data file formats. R also allows saving images of the R *environment* to a compressed file. However, absolute beginners often struggle to grasp the notion of R environment. In our workshops, we conveyed this notion with the aid of an imagery of a traditional data analysis work space (see Figure 2). When one starts a new job, they may begin their work at an empty desk. As they proceed with their work, they would populate their work environment with objects that may store data, e.g., taskpads or other office stationery. Similarly, when one starts a new R session, they begin with an empty R environment. As they progress with analysis, they create R objects that have a name label and contain data. These are visible in the R environment. A smart feature of R is that it allows users to save the environment as a file and load at a later time in a new R session, which is analogous to packing up the books or printouts at one’s desk when a session of work ends and rearranging them on the desk whenever needed again.

**Figure 3.**
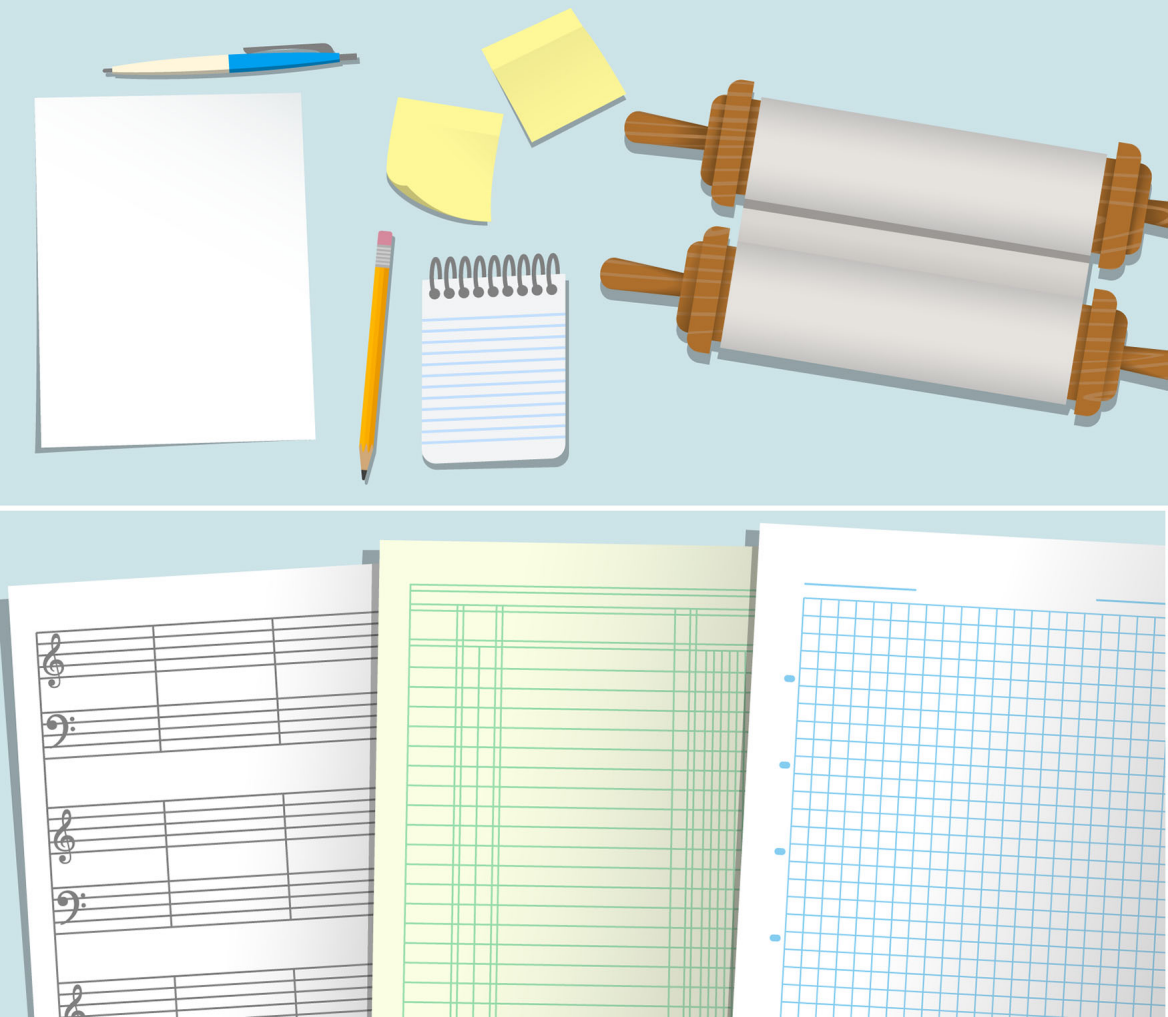
R objects have physical analogues. Diverse data structures are used in R to store different kinds of data. This is analogous to the use of various objects as shown in the illustrations to store data in non-digital life.

**Handling of diverse and complex use cases.** A typical session of data analysis might involve feeding data that exists in paper or digital media such as text/image files to a computing device and saving desired results in a convenient format. R supports diverse file formats for both the input and output, which is a substantial advance over ordinary calculators that typically accept and output numeric values only. Further, there are flexible options to organize or structure data within an R session, and a number of functions to support sampling from existing data and reorganizing data in meaningful ways. In our workshops, when we were introducing data types and structures in R, and the functions called head, tail, colnames, etc. that display the first few entries, last few entries, column names (if applicable) of R data objects, respectively, we found the trainees wondering what the point of learning about these was. Although we were asked an explicit question about this only once out of ten sessions, we suspect that many more beginners may benefit from a motivation when discussing such concepts, which we communicated as follows. In a typical calculator, the allowed data type is predominantly single numeric value. In contrast, R can handle data structured in various formats. For example, in real life, sometimes our needs of storing data would be better served by a sticky note than by a notebook, or by a printer paper, etc. (see Figure 3). Similarly, in R, our needs are sometimes better served by tabular structures, at other times by an array, or at other times by a list structure. In fact, a smart feature of R is that package developers may define their own data structures for special purposes, e.g., DGElist in the *edgeR* package, SeuratObject in the *Seurat* package, which are analogous to special-purpose formats, e.g., lab notebooks, sheet music, ledger, etc. When handling a notebook containing data, one flips through the pages in many ways to examine how the data is organized. Similarly, functions such as head, tail, colnames, etc. enable us to examine R objects.

**Support for literate programming.** A popular advice is to treat a program as a piece of literature, addressed to human beings rather than to a computer. ^23^ Such practice facilitates reproducible research and enables open access, which are easily achievable goals in the digital age. To this end, R provides the option to include text and analysis results along with blocks of code in the same document, e.g., as RMarkdown documents. Additionally, comments and use of indentation in scripts make R documents easy for humans to read. Once again, in an age of smart devices, this is a desirable way to write and think about programs. Further, it has been suggested that programs and algebra play analogous roles in quantitative sciences. ^24^ Highlighting these points may equip beginners with a framework to think about R scripting.

## Introductory workshops should seamlessly integrate an understanding of the elements of R with participatory live coding

We delivered an understanding of the R system using analogies described above, and strove to present them in seamless integration with participatory live coding. In feedback, our trainees described participatory live coding as one of the best parts of our workshops. We conducted our workshops in person and via videoconferencing to support remote learning. To design the workshops, we studied tips suggested in literature by other instructors. ^25, 26^ In particular, we found it helpful to assess student learning periodically by asking them to guess what a line of code might do and to suggest code for some of the tasks. However, we kept our workshops informal and participation voluntary. Our goal in doing so was to accommodate individuals who are looking to learn passively by being present in workshops while also attending to other work, e.g., responding to emails. In our workshops, presence of passive learners did not interfere with the attention of other students actively following instruction. We allowed attendees to interrupt with questions at any time including by unmuting themselves in remote sessions. Our goal was to create a relaxed, no-judgement workshop environment where participants can feel free to ask any question and do as they please.

The feedback revealed that the choice of data for practice during the workshop is an important consideration. Most trainees prefer to work with a data type that they have experience with and is relevant to their research. Since our audience was almost all biomedical researchers, we switched to using a bulk RNA-seq counts matrix after using the *Iris* flower data ^27^ for initial iterations of our workshop. For general audiences, we recommend pre-workshop surveys to identify datasets that most attendees might find interesting. In some of our workshops, we found it helpful to share a summary of a pre-workshop survey with the attendees. Awareness of the class composition helped with making the students with somewhat more advanced backgrounds (e.g., experience with other programming languages) patient while we answered questions from absolute beginners.

## Introductory workshops should provide specific examples of what students can do after the workshop

Independent surveys have found that retention of skills taught in short workshops tends to be poor. ^9^ Hence, it is important that introductory workshops give specific examples of how students can integrate R in their research practice. In our workshops, we recommended that students ask their computational collaborators (if any) for code, or download them for published papers they may read. At the very least, they should study them as part of their research, which can teach them about new functions and programming practices. Additionally, they could ask their collaborators for guidance in making minor modifications to the code for exploratory analysis. We browsed examples of RMarkdown documents and scripts available online to show that they can be studied the same way as research literature with code chunks having the same role as algebraic equations, only easier to understand because R function names are in many cases abbreviations of English words.

Additionally, it is important for beginners to have an awareness of things that they should explore in their own time. For example, in a four hour long introductory workshop, we could not cover concepts such as conditional statements, loops, and hypothesis tests via participatory live coding. We dedicated the last 5-10 minutes to discussing these and browsing the R graph gallery. ^28^ These provide students with a concrete direction to continue learning and exploring R after the workshop.

## Discussion and conclusion

A major factor that shaped the thought process underlying our workshop design was the duration of workshop. This was set to four hours based on our assessment of availability of postgraduate learners in our community, the other workshops that we teach throughout the year, and our own research load. To provide something of practical utility to absolute beginners with advanced needs in this short time frame, we came up with a way to communicate the key ideas that will serve as a mental framework to guide self-learning after the workshop. However, this can have an unintended effect of making beginners feel that using R is as simple as using a calculator, i.e., it is not important to look under the hood for how R functions are processing data. Consequently, it is important to warn the beginners to be cautious and check the function documentation and relevant literature to ensure that the methods implemented in the function are suitable for their purpose. If the workshop duration permits, instructors could consider demonstrating cases of undesirable outcomes due to uninformed use of statistical or other functions. In feedback, some of our attendees suggested that the duration could be longer than four hours, include more advanced statistical analysis and visualization with packages such as *ggplot2*, ^29^ and allow time for practicing on their own but in the presence of instructors. We found it challenging to include these in our introductory workshop and conduct separate workshops on these topics. Other instructors may find it beneficial for their communities to conduct workshops over longer duration, and include these components in their design.

Besides providing analogies to enhance teaching effectiveness, one of our goals in writing this manuscript is to expose the conceptual chasm that exists between the instructors of introductory programming workshops and absolute beginners. To be effective, the instructors should be aware of the questions that beginners may have on their minds but may not ask. Many of our workshop attendees prefaced their questions with a statement such as “sorry for a stupid question”, which suggests that students have to overcome a sort of guilt feeling before they ask a question. In our opinion, all of their questions are valid and should be answered to welcome beginners into the fold of programming. Additionally, since our workshops were free-of-cost, our experience has been that there are always at least some individuals who stroll into a workshop without prior planning or any study on their own. Even though there are professional benefits from learning to program, instructors are competing with other online platforms for attention, which is only made more tough when the students do not feel a sunk cost in the form of payment for attending workshops. Yet, demanding payment for training from individual students can slow down the progress to a future when all bioscientists will be computational bioscientists. Hence, several institutions including ours sponsor free data science training workshops for their community members and/or facilitate access to online courses. In this article, we proposed that introducing R (or other languages for data analysis) as a smart version of calculators can capture the attention of absolute beginners and make learning to program feel like switching from first-generation mobile phones to smartphones—a logical and pleasant move to an intuitive way to interface with technology. For other instructors, when designing their workshops, important considerations should be to tell the story the way they have it in their head and customize for their audience. Whether they use the analogies presented in this article or not, a workshop should not be fragments of code presented in succession. It is important to have one unifying theme that is easy to remember and focuses on the R system in general instead of the specific data being analyzed. For our purpose, our proposition that R is a smart version of calculators worked well.

## Data availability

No data is associated with this study.
