# Supplementary material for: Introducing R as a smart version of calculators enables beginners to explore it on their own
Source: F1000Res. 2022 Jun 21;10:859. Originally published 2021 Aug 26. [Version 2] doi: 10.12688/f1000research.54685.2 (PMC8976183; doi:10.12688/f1000research.54685.2)
Supplement: Peer review history

# Peer review: Reviewer response for version 2

**DOI:** 10.5256/f1000research.135007.r141468

**Published:** 2022-07-06

**Authors:** Lex Nederbragt

**Affiliations:** 1 Department of Biosciences, University of Oslo, Oslo, Norway

Thanks to the authors for the revised version! I have no further comments.

Is the topic of the opinion article discussed accurately in the context of the current literature?

Partly

Are arguments sufficiently supported by evidence from the published literature?

Partly

Are all factual statements correct and adequately supported by citations?

Yes

Are the conclusions drawn balanced and justified on the basis of the presented arguments?

Yes

Reviewer Expertise:

Bioinformatics, Genomics, Education

I confirm that I have read this submission and believe that I have an appropriate level of expertise to confirm that it is of an acceptable scientific standard.

# Peer review: Reviewer response for version 1

**DOI:** 10.5256/f1000research.58191.r128422

**Published:** 2022-04-05

**Authors:** Rayna M Harris

**Affiliations:** 1 Department of Population Health and Reproduction, University of California Davis, Davis, CA, USA

The opinion article "Introducing R as a smart version of calculators enables beginners to explore it on their own" provides practical advice for teaching R to trainees in the biological sciences. Designing workshops for beginners is challenging because trainees have diverse skills and learning objectives. The authors provide multiple analogies that could be used in an "Introduction to R workshop" to make R more accessible to beginners. The figures bring the analogies to life and could easily be inserted into a slide deck for a workshop (with permission) to teach these concepts. I think the article provides a unique perspective on teaching that is relevant to anyone teaching computational tools to diverse audiences.

 Like Reviewer 1, I would support the indexing of the opinion article in its current state. I do, however, have a few comments and questions.

 I noticed that you cited the iris dataset but not the RNA-seq data used for your workshop. A citation for that data would be useful to the reader and appreciated by the data generators. Also, do you teach edgeR and DESeq2 to beginners, or was this provided as an example? I think a citation here would be good to show how often these tools are being taught and used.  

 In the data availability section, it says that there is no data associated with this study. Would it be possible to link some of the files used in previous workshops (such as R scripts, slides) that could be used by instructors wishing to incorporate these suggestions into their classrooms? Also, you mentioned showing the results of pre-workshop surveys at the beginning of a workshop. Can you share those data summaries and/or survey questions (or is that private)? This could provide a nice overview of the target audience's field of study and prior knowledge.

Is the topic of the opinion article discussed accurately in the context of the current literature?

Yes

Are arguments sufficiently supported by evidence from the published literature?

Yes

Are all factual statements correct and adequately supported by citations?

Partly

Are the conclusions drawn balanced and justified on the basis of the presented arguments?

Yes

Reviewer Expertise:

Genomics

I confirm that I have read this submission and believe that I have an appropriate level of expertise to confirm that it is of an acceptable scientific standard.

# Response

**Published:** 2022-06-10

**Authors:** Krishna Choudhary

**Affiliations:** University of California San Francisco Medical Center, USA

Thank you for the positive assessment of our submission and the suggestions. In the revision, we have cited the RNA-seq data we used, and the articles describing DESeq2 and edgeR. These libraries were covered in separate workshops later in the series and were indeed just provided here as examples. We have also provided a link to the workshop materials. We did not save the pre-workshop surveys, but the large majority of our workshop attendees self-identified as beginners to R scripting and programmatic data analysis, and consisted of graduate students and postdoctoral scholars pursuing biomedical research. Occasionally, faculty also attended the workshops.

# Peer review: Reviewer response for version 1

**DOI:** 10.5256/f1000research.58191.r119622

**Published:** 2022-04-01

**Authors:** Yasha Hasija

**Affiliations:** 1 Department of Biotechnology, Delhi Technological University, Delhi, New Delhi, India

The authors of this opinion article have discussed the analogy of introducing the R programming language as a smart version of calculators in order to present the concept in a more simple and understandable manner. The article explains various analogies that can be derived while teaching the R programming language in a clear and concise manner. As a smart calculator R employs programmed functions, which are analogous to the calculator's push-button operations. These functions can be created through logical or arithmetic programming or downloaded from internet repositories such as CRAN and Bioconductor. Some of the advantages of R over traditional calculators have been discussed in terms of new interfaces via programming, Internet-enabled updates to available options via packages, secondary memory for long-term storage, and handling of diverse and complex use cases.

 In addition, the authors have tried a few methods for introducing R to beginners via a webinar. They presented a concept of R programming using the analogies mentioned above, attempting to integrate them seamlessly with a participatory live coding webinar. The program was scheduled for four hours, and based on their evaluation, newbies were convinced that R is as simple to use as a calculator. While they were unable to cover conditional statements, loops, and hypothesis testing in a four-hour webinar. Overall, the authors addressed in this article how introducing R as a smarter version of calculators can spark a new interest in complete beginners. However, presenting an exploratory analysis of the feedback data may have made the article more conclusive.

Is the topic of the opinion article discussed accurately in the context of the current literature?

Yes

Are arguments sufficiently supported by evidence from the published literature?

Yes

Are all factual statements correct and adequately supported by citations?

Yes

Are the conclusions drawn balanced and justified on the basis of the presented arguments?

Yes

Reviewer Expertise:

Computational Biology, Bioinformatics

I confirm that I have read this submission and believe that I have an appropriate level of expertise to confirm that it is of an acceptable scientific standard.

# Response

**Published:** 2022-06-10

**Authors:** Krishna Choudhary

**Affiliations:** University of California San Francisco Medical Center, USA

We thank the reviewer for their thoughtful summary of our article, and suggestion for further analysis.

# Peer review: Reviewer response for version 1

**DOI:** 10.5256/f1000research.58191.r126471

**Published:** 2022-03-23

**Authors:** Lex Nederbragt

**Affiliations:** 1 Department of Biosciences, University of Oslo, Oslo, Norway

This opinion piece suggests motivating researchers to use R by introducing the tool as an advanced calculator first. By making analogies to things new users are already familiar with, the authors aim to lower the barrier for getting started. The authors describe a 4-hour workshop where this approach is used successfully.

 I like the use of these analogies and enjoyed reading the article. I would suggest others also try this approach for other programming languages or computational tools. Often researchers will come to a workshop to learn a tool they have heard about and that seems to be important for their work, but are uncertain whether they will be able to master it. Connecting the tool to something familiar makes for a smoother introduction.

 I would support the indexing of the opinion article in its current state. I do, however, have a few comments and suggestions.

 First, although not strictly necessary, the article indirectly refers to concepts from the sciences of learning (e.g., cognitive psychology). For example, research has shown that connecting new knowledge to prior existing knowledge helps with learning. Another useful concept would be that of mental models: the authors are first providing a mental model of R close to the familiar one of a calculator, then expand on it to show the power of R as an analysis tool beyond calculations. A third example is that to "assess student learning periodically" is known as 'formative assessment', a way (for instructors and learners!) to check that learning actually has happened. Anchoring the approach taken by the authors to these phenomena from the learning sciences would strengthen their validity.

 Some more specific comments:

 Could the authors include a bit more on the intended audience of their workshops, for example how the workshop was announced? The reader would benefit from a brief explanation of what Participatory Live Coding is.

 In the section "Handling of diverse and complex use cases", where data structures and tail, head, colnames are discussed: to me, using the analogy of a table of data in a spreadsheet program (Excel, Google Sheets) came up as a useful way to introduce R data objects. Have you considered using that?

 I found the section "Support for literate programming" a bit confusing. I agree that the use of RMarkdown and the creation of computational notebooks is a useful aspect of choosing a tool like R, but the arguments in this section are a bit vague and I wonder whether most readers will follow them. For example, I do not understand the connection to 'smart devices' or the reason for including the next sentence about the comparison of programs and algebra.

 Finally, when reading the article, I could not help thinking about how nicely this fits in with the 'philosophy' of The Carpentries, the international volunteer organization teaching coding and data analysis skills to researchers ( https://carpentries.org). Are the authors aware of The Carpentries? The suggestions from this opinion article could be tried out in one of their workshops as well.

Is the topic of the opinion article discussed accurately in the context of the current literature?

Partly

Are arguments sufficiently supported by evidence from the published literature?

Partly

Are all factual statements correct and adequately supported by citations?

Yes

Are the conclusions drawn balanced and justified on the basis of the presented arguments?

Yes

Reviewer Expertise:

Bioinformatics, Genomics, Education

I confirm that I have read this submission and believe that I have an appropriate level of expertise to confirm that it is of an acceptable scientific standard.

# Response

**Published:** 2022-06-10

**Authors:** Krishna Choudhary

**Affiliations:** University of California San Francisco Medical Center, USA

Thank you for the positive assessment of our submission and the thoughtful suggestions. Per your suggestions, we have:

- added text and references to mental models and formative assessment;
- described the intended audience and defined “participatory live coding”;
- included spreadsheet programs as another analogy;
- a citation to Software Carpentry.
